# The Role of Exosomes in Pancreatic Cancer

**DOI:** 10.3390/ijms20184332

**Published:** 2019-09-04

**Authors:** Bin Lan, Siyuan Zeng, Robert Grützmann, Christian Pilarsky

**Affiliations:** Department of Surgery, Universitätsklinikum Erlangen, Krankenhausstraße 12, 91054 Erlangen, Germany (B.L.) (S.Z.) (R.G.)

**Keywords:** pancreatic cancer, exosomes, chemoresistance, biomarker

## Abstract

Pancreatic cancer remains one of the deadliest cancers in the world, as a consequence of late diagnosis, early metastasis and limited response to chemotherapy, under which conditions the potential mechanism of pancreatic cancer progression requires further study. Exosomes are membrane vesicles which are important in the progression, metastasis and chemoresistance in pancreatic cancer. Additionally, they have been verified to be potential as biomarkers, targets and drug carriers for pancreatic cancer treatment. Thus, studying the role of exosomes in pancreatic cancer is significant. This paper focuses on the role of exosomes in the proliferation, metastasis and chemoresistance, as well as their potential applications for pancreatic cancer.

## 1. Introduction

Pancreatic cancer (PC) is the seventh most common malignancy, and the fourth and sixth leading cause of cancer-related death in the United States and China, respectively [1]. Regrettably, the 5-year survival rate for patients merely takes up 5–10%, and the median survival time is 5–6 months after diagnosis [2,3]. The majority of the patients with pancreatic cancer fail to develop prominent symptoms before reaching the advanced stage of the disease [1,2], which caused 432,242 new deaths worldwide in 2018 [4]. The poor prognosis of pancreatic cancer is typically caused by various factors, including poor detection rates at the initial stages, the high risk for distant metastasis, and disappointing surgical and chemotherapy outcomes. The CA19-9 antigen test that is currently utilized is not sufficient to diagnose PC with high sensitivity and specificity [5,6]. Surgical resection is taken as the only potential curative therapy for pancreatic cancer [7], while 5-fluorouracil/leucovorin with irinotecan and oxaliplatin (FOLFIRINOX), gemcitabine combined with nanoparticle albumin-bound paclitaxel (nab-paclitaxel) constitute the first-line chemotherapy for patients with advanced pancreatic cancer [8,9,10,11,12,13]. Unfortunately, surgical resection is usually followed by short-term recurrence, and the effectiveness of chemotherapy drugs is often plagued by chemoresistance, aggravating the prognosis of patients with metastatic disease [14,15,16,17]. Therefore, early diagnosis, curbing metastasis, and exploring effective treatment methods are the urgent issues in the treatment of pancreatic cancer.

In the preceding decade, exosomes have attracted worldwide attention among researchers because of their special roles in multiple facets of cell activity, especially in the progress of cancer [18,19,20,21]. It is widely accepted that exosomes are membranous vesicles with lipid bilayer membranes ranging in diameter from 40 to 100 nm, and being secreted by multiple cell types and cancer cells [22,23], containing functional biomolecules (including lipids, proteins and nucleic acids). They participate in many physiological processes, such as immune response, antigen presentation, protein and RNA transport [19]. Having been demonstrated to be signaling vehicles for intercellular communication between the tumor and contiguous organs, exosomes were highlighted as cell-to-cell communication tools and mechanisms of molecular transfer in recent years [16,18,20,24]. In this review, the research status and development in the field of exosomes will be briefly introduced, and special attention will be paid to exosomes in pancreatic cancer, chemoresistance, and its potential application in pancreatic cancer.

## 2. Biological Features of Exosomes in PC

### 2.1. Definition, Morphology and Composition of Exosomes

**Figure 1:**
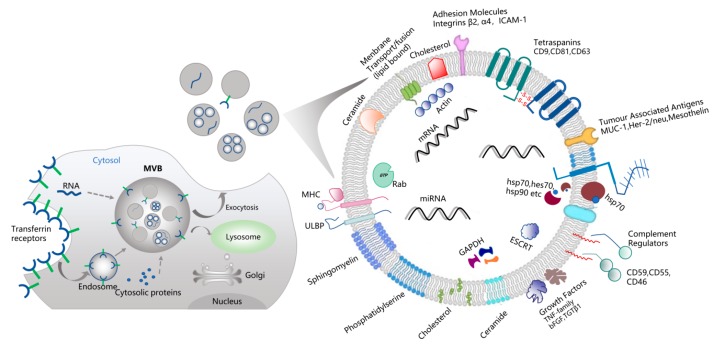
Exosomes biological features. Exosomes are vesicles of endocytic origin. After the plasma membrane sprouts inward to form the early endosomes, the multivesicular bodies (MVBs) further sprouts inward to produce intraluminal vesicles (ILVs), MVBs fuse with the plasma membrane, releasing exosomes into the extracellular space. Exosomes are mainly consisted by proteins, lipids and nucleic acids. Nucleic acids include microRNA (miRNA), transfer RNA (tRNA), ribosomalRNA (rRNA), messenger RNA (mRNA), and non-coding RNA (ncRNA). Exosomes also contain endosome-specific tetraspanins (CD9, CD63, CD81) on their membrane surfaces. major histocompatibility complex, MHC; UL16 binding protein, ULBP; hot shock protein, hsp; ras-related gtp-binding protein, Rab; endosomal sorting complex required for the transport, ESCRT.

Extracellular vesicles (EVs) include exosomes, microvesicles, ectosomes, apoptotic bodies, and oncosomes, and they are categorized based on their sizes and biogenesis mechanisms [25]. Exosomes were originally introduced by Johnstone et al. in 1987 during the culture of sheep reticulocytes in vitro [23]. In the document, exosomes are generally depicted as having diameters less than 100 nm, while microvesicles are considered to be larger than 100 nm [26]. The classical definition of exosomes is that they originate from the endosomal compartment by fusing multivesicular bodies (MVBs) with the plasma membrane, while microvesicles and exosomes are thought to sprout directly from the plasma membrane [23,26,27]. The initial stage of formation of exosomes is that the plasma membrane sprouts inward to form an early endosome. In the maturation of the endosome, the endosomes germinate inward in a limited area to form nano-sized vesicles, resulting in MVB that contain intraluminal vesicles (ILVs) [23,28,29], which contain cytoplasmic components including nucleic acids and soluble proteins. When MVBs are formed, the ILVs will be released to the extracellular environment by fusing the MVBs with the plasma membrane (Figure 1) [25,30]. The secreted exosomes function in fundamental pleiotropic biologic processes through directly interacting with their transmembrane proteins or lipid ligands with cell surface receptors, then delivering the cytosolic proteins and nucleic acids into recipient cells through membrane fusion [30]. Due to this characteristic, they might act as postmen in cell interactions and might be pivotal in the occurrence and development of diseases, including tumor progression, metastasis, and promotion of immune escape [21,31,32].

Exosomes have been isolated from multiple body fluids like blood plasma [33,34,35], serum [36,37], urine [38], breast milk [39], and saliva [40,41]. Exosomes mainly consist of proteins, lipids and nucleic acids. Some proteins, lipids and nucleic acids are enriched in specific exosomes, while other proteins and lipids are omnipresent in all exosomes [33,34,35]. It is known that lipids, such as sphingomyelin, cholesterol, ceramide, and phosphatidylserine are enriched in most exosomes. Nucleic acids include microRNA (miRNA), messenger RNA (mRNA), transfer RNA (tRNA), ribosomalRNA (rRNA), and non-coding RNA (ncRNA) [19,23,34,35]. Exosomes also contain endosome-specific tetraspanins (CD9, CD63 and CD81), and MVBs biogenesis-related proteins (TSG101, Alix) on their membrane surface [42,43]. In addition to the characteristics of membrane markers, the molecular content of exosomes can also change profoundly according to the original cell type and physiological conditions.

### 2.2. Isolation of Exosomes

Most methods adopted to isolate and characterize exosomes are based on physical and chemical properties. To date, various approaches, like ultracentrifugation (UC), size exclusion chromatography, magnetic activated cell sorting (MACS), membrane filtration and commercial kits have been used to separate exosomes from cell culture medium and body fluids. Different methods can be used according to the requirements of the experiment. However, the majority of published research on exosomes have utilized UC for exosome isolation. Although no uniform standard has been established to isolate and identify exosomes at present, the above methods have been proved effective in different literatures, and the combination of the two methods is usually conducted to provide the extracted exosomes with better abundance and purity. The International Society for Extracellular Vesicles (ISEV) has offered authoritative guidance for EV isolation and purification and updated the guidelines recently [25,27]. Exosome detection can be realized by transmission electron microscopy (TEM) and nanoparticle tracking analysis (NTA), while western blotting and flow cytometry can be used to analyze and detect exosome markers. The markers employed to analyze exosome include tetraspanins (CD9, CD63 and CD81), endosomal sorting complex required for the transport (ESCRT)-associated proteins (TSG101 and ALIX), cytoplasmic proteins (HSP70 and HSP90), adhesion molecules (integrins), and membrane transport and fusion proteins (Annexins) (Figure 1) [19,26].

## 3. Role of Exosomes in PC

### 3.1. Exosomes Regulate PC Cell Proliferation

Exosomes contain abundant proteins, lipids, and nucleic acids, which are pivotal in the interactive transmission of cell to cell information exchange, while exosomes of different origins are distinct in regulating the proliferation of PC cells. Cancer-related fibroblasts (CAFs) which are developed from bone marrow-derived mesenchymal stem cells (MSCs) are inextricably linked with aspects of proliferation in PC [44]. Recent studies have shown that exosomes released by gemcitabine-treated CAFs increased the proliferation and survival of PC cell lines. This was, in some part, due to the enhanced level of miR-146a and Snail in recipient cells. Restraint of exosome secretion from CAFs decreased the proliferation and survival of PC cells [5]. Recently, bone marrow mesenchymal stem cell (BMSC)-derived exosomes in pancreatic cancer have attracted extensive attention; with the aim to elucidate how non-tumor derived exosomes will impact the proliferation, invasion and apoptosis of pancreatic cancer cell lines, along with tumor growth and metastasis by means of medium transfer. The study demonstrated that the over-expressed microRNA-126-3p from the exosome of BMSCs inhibits the proliferation, invasion and metastasis of pancreatic cancer cells and promotes apoptosis in vitro and in vivo by targeting down-regulation of a disintegrin and a metalloproteinase-9 (ADAM9) [45]. More interestingly, moderate malignant pancreatic cancer cell lines enhance their capacity to proliferate, migrate, and invade by ingesting exosomes derived from highly malignant pancreatic cancer cells [46]. The same group also indicated that a zinc transporter ZIP4 is the most up-regulated exosomal protein in the cell lines of moderate malignant pancreatic cancer, which directly promotes the growth of recipient cells and is expected to become a new diagnostic marker for patients with pancreatic cancer. Furthermore, the highly concentrated exosomes derived from tumor-associated stroma (TAS) cells have the ability to impart tumor inhibition on adjacent pancreatic ductal adenocarcinoma (PDAC) cells by delivering miRNAs likemiR-145 [47]. These in vitro and in vivo studies indicated that whether exosomes are favorable or detrimental to cell proliferation is decided on cell type and cell features. Different sources of exosomes have different effects on PC cell proliferation, or even the opposite, which needs further clarification and in-depth study (Table 1).

### 3.2. Exosomes Promote PC Invasion and Metastasis

Pancreatic cancer is characterized by its high invasiveness and metastasis, which is a major cause of treatment failure. Through invasion and migration, cancer cells can be exuded to distant sites and colonized in secondary tissues and organs. Exosomes modulate PC invasion and metastasis due to their regulatory impacts on pancreatic cancer cells and the tumor microenvironment. Exosomes have been thought to be involved in the proliferation of cancer cells, and plenty of research groups have pointed out how tumor-derived exosomes participate in invasion and metastasis [20,21,32]. Recent evidence suggests that exosomes may promote cancer invasion and metastasis through regulating stromal cells, reshaping the extracellular matrix (ECM) and stimulating angiogenesis. In particular, exosomes can enhance directional cell motility via ECM components, like fibronectin, to offer a substrate benefiting cell adhesion and increasing cell speed [48]. Recent research indicated that hypoxic exosomes derived from pancreatic cancer cells could activate the phosphatase and tensin homolog (PTEN)/phosphorinositol 3-kinase (PI3K) gamma signaling pathway, followed by stimulating macrophages to the M2 phenotype in a hypoxia inducible factor 1 or 2 subunit alpha (HIF1a or HIF2a)-dependent way, which facilitates the invasion, migration and epithelial-mesenchymal transition (EMT) of PC cells. Further investigation demonstrated that miR-301a-3p was prominently displayed in hypoxic pancreatic cancer cells and enriched in their exosomes. The PC cells metastatic ability increased after it was cocultured with miR-301a-3p up-regulated macrophages or treated with hypoxic exosomes [49]. Another in vivo study found that tumor-secreted exosomal miR-222 can stimulate AKT by constraining protein phosphatase 2 regulatory subunit Balpha (PPP2R2A) expression, and thus inducing p27 phosphorylation and cytoplasmic p27 expression to promote invasion, metastasis and survival [50]. Moreover, the EMT is supported by CD151−/tetraspanin 8-competent exosomes, which leads the distinction of non-metastatic PC cells to a motile phenotype [51]. Apart from enhancing the invasiveness of cancer cells, exosomes can also promote angiogenesis by transferring proteins (such as VEGF and TGF-β) and RNA, thus establishing a metastatic microenvironment. In brief, exosomes derived from tumor cells or the tumor microenvironment indeed function positively in invasion and metastasis through the help of different molecules (Table 1).

### 3.3. Exosomes Participate in the Formation of Chemoresistance in PC

In the past decade, gemcitabine was widely used in chemotherapy and served as a first-line drug to treat advanced pancreatic cancer [10,12]. Although gemcitabine and other drugs bring benefits to pancreatic cancer patients, the advance of chemoresistance to gemcitabine severely limits the effectiveness of chemotherapy. Exosomes are an important tool for intercellular material and information exchange in that they regulates the microenvironment of tumors by transferring gene and protein signals between cells, thus mediating the angiogenesis, differentiation, apoptosis and metastasis of tumors. Building on accumulating evidence, exosomes and their microRNAs and proteins may act as cell-to-cell communicators in enhancing chemoresistance in various cancers, such as lung cancer [52], breast cancer [53], leukemia [54,55], prostate cancer [56], glioblastoma [57,58], gastric cancer [59,60,61], and PC [14,15,16,17].

CAFs are inherently insensitive to gemcitabine and vital in the development of chemoresistance in tumor cells. Once exposed to gemcitabine, pancreatic fibroblasts significantly enhance the release of Snail and miR-146a through exosomes, which can be absorbed by recipient epithelial cells. Therefore, CAF-derived exosomes promote proliferation and chemoresistance [5]. In addition, CAFs can also transfer miR-21 to cancer cells through exosomes, inducing chemoresistance by binding apoptotic peptidase activating factor 1 (APAF1) or activating the phosphorinositol 3-kinase (PI3K)/AKT signaling pathway [62].

Exosomes can regulate drug resistance of cancer cells at the gene level through paracrine action. Therefore, there may be a group of microRNAs that transfer chemoresistance phenotypes to sensitive cells by changing cell growth, as well as causing anti-apoptotic processes. After incubation with gemcitabine, the expression of miR-155 was up-regulated in PC cells, and miR-155 was transferred to PC cells through exosomes. MiR-155 promotes chemoresistance in PC cells through the anti-apoptotic pathway, and inhibits deoxycytidine kinase (dCK) [16]. In addition, the over expression of miR-155 up-regulated the synthesis and secretion of exosomes, as well as the content of miR-155 in exosomes, forming a positive cycle regulating drug resistance [16,63]. Furthermore, exosomes conferred chemoresistance to PC cells by enhancing the detoxification of reactive oxygen species (ROS) through the expression of superoxide dismutase 2 (SOD2) and catalase (CAT) [16]. Exosomes isolated from chemoresistant PANC-1 cells can also enhance the gemcitabine resistance to less chemoresistant PC cell lines MIA PaCa-2 and BxPC-3 via transferring Ephrin type-A receptor 2 (EphA2) [64]. Taken together, exosomes from PC or tumor microenvironments promote chemoresistance by regulating proteins, related genes, RNAs and signaling pathways. However, extensive and in-depth studies are required to further explain how exosomes mediate and transmit related chemoresistance in PC (Table 1).

## 4. Exosomes as Diagnostic and Prognostic Biomarkers of PC

The poor prognosis for pancreatic cancer is mainly a result of the lack of specific symptoms in the early stages of pancreatic cancer, and as a tumor marker for clinical routine screening, the specificity and sensitivity of CA19-9 are relatively low, so many patients have already reached an advanced stage at the time of diagnosis [5,6]. Thus, it is urgent to search for new biomarkers that can detect early lesions that are highly sensitive and specific, and can differentiate between PC from healthy lesions and benign pancreatic diseases. Up to now, people have been working on the development of sensitive diagnostics tools to improve the early detection of pancreatic cancer by identifying exosomal signs related to pancreatic cancer. As discussed above, exosomes extracted from cancer cells are enriched with proteins, mRNA, and miRNA, and exhibit stability and abundance in various biological fluids [65,66].

The potential of exosome proteins and RNAs as biomarkers for diagnosis and prognosis indicates increasing application and attention (Table 2). A recent study evaluated the expression patterns of four pancreatic cancer-related miRNAs (miR-21, miR-155, miR-17-5p and miR-196a) in circulating exosomes as biomarkers. Serum miRNA was detected by RT-PCR in 49 patients, including 22 with PCs, 6 with benign pancreatic tumors, 6 with chronic pancreatitis, 7 with ampullary carcinomas and 8 healthy controls. Compared with control groups, the expression profile of miR-17-5p and miR-21 was remarkably enhanced in PC patients, with sensitivity and specificity of 72.7% and 92.6% and 95.5% and 81.5%, respectively. The increased expression of miR-17-5p in metastasis and advanced PC indicates that miR-17-5p is a possible biomarker for unresectable patients [67]. Recent research has focused on elucidating differential miRNA profiles for pancreatic cancer by comparing miRNA expression in pancreatic cancer patients and healthy controls [68]. The next generation sequencing and qRT-PCR analysis of exosomal microRNAs from PC are important tools to identify biomarkers for diagnosis and prognosis of pancreatic cancer. There were similar results in studies on miR-10b [69], miR-550 [70], miR-196a, miR-1246 [71], and miR-451a [72]. These results presented increased levels in exosomes isolated from pancreatic cancer cells, indicating that they can function as early biomarkers for the diagnosis of PC.

Additionally, exosomal proteins are equally important in PC diagnosis. Melo et al. reported that glypican-1 (GPC1) was specifically enriched on PC exosomes. Levels of GPC1+ circulating exosomes were significantly enhanced in PC patients compared to the healthy group, indicating that the sensitivity and specificity of GPC1+ circulating exosomes in diagnosing PC were both 100%, while levels of CA19–9 in serum cannot distinguish patients with PC from those with benign pancreatic disease [37]. Furthermore, levels of GPC1+ circulating exosomes correlate with tumor burden and the survival of PC patients [37,73]. Macrophage migration inhibitory factor (MIF) is highly expressed in pancreatic cancer-derived exosomes, and its inhibitory effect can prevent the formation and metastasis of pancreatic cancer before the formation of liver metastasis. Compared with patients without progression of pancreatic tumors, MIF in patients with stage I PC who later developed liver metastasis gained significantly, indicating that exosomal MIF plays an important role in liver metastasis and may be a prognostic marker for predicting liver metastasis [74]. Another recent research indicated that exosomal integrins α6β4 and α6β1 were related to lung metastasis, while exosomal integrin αvβ5 was connected with liver metastasis. The amounts of integrin αvβ5 were profoundly improved in exosomes isolated from PC with liver metastasis compared with no distant metastasis. The mechanisms demonstrated that integrin αvβ5 uptake by recipient cells was able to result in activating Src phosphorylation and upregulation of the pro-inflammatory S100 gene. Further clinical data suggest that exosomal integrins could be prognostic biomarkers used to predict distance metastasis [75].

The majority of these studies focus on the difference of exosomes expression between pancreatic cancer patients and healthy people, and the evaluation of PC exosomes as a biomarker for diagnostic and prognostic purposes. Nevertheless, additional studies may still be required to validate whether exosomes can be used to evaluate the therapeutic effects and response of pancreatic cancer. Exosomes still have many obstacles in clinical application as a potential biomarker, due to the intricate procedures in isolation and purification.

## 5. Emerging Role of Exosomes in PC Therapy

Due to their key roles in cellular signaling and transportation, the development and biological functions of exosomes have drawn accumulating public attention. Apart from the giant potential in early diagnosis of pancreatic cancer, the unique lipid bilayer structure and specific surface proteins make it possible to use exosomes as nanoparticle carriers to transport drugs, macromolecular nucleic acids, and proteins. A growing body of evidence suggests the safety and efficacy of exosomes in treating pancreatic cancer. During the past few years, exosome-based therapies for cancers have been developed, especially in drug delivery, due to the permeability of the exosomal membrane, allowing it to easily pass through the blood-brain barrier. Compared with other nanoparticle transport means, the exosomes have no toxic side effects and low immunogenicity. Integrating the above advantages, exosomes are an emerging means and an ideal candidate for pancreatic cancer treatment.

### 5.1. Exosomes as Therapeutic Targets

As described above, exosomes are vital in pancreatic cancer progression and drug resistance formation. Therefore, the current treatment strategy mainly lies in inhibiting the production of cancer cell exosomes and blocking the uptake of specific exosomes by receptor cells. However, there is still a lack of clear understanding of the production and secretion pathway of exosomes. Ostrowski et al. identified the key Rab GTPase for exosome secretion by RNA interference (RNAi) screening while indicating that this process was mainly regulated by Rab27a and Rab27b [76]. GW4869, an inhibitor of nSMase2, is a widely used exosomes release inhibitor which significantly inhibits exosome production [29,77]. As mentioned above, CAF is naturally resistant to gemcitabine and can transmit chemoresistance. However, when the exosome release inhibitor GW4869 was added, the chemoresistance of PC cells transferred by GEM-exposed CAF was eliminated and the survival rate of PC cells was significantly reduced [5]. After transfection of PC cells with siRAB27B, the number of exosomes was significantly reduced, resulting in a significant decrease in miR-155 induced GEM resistance [63]. Another effective strategy for exosome-targeted therapy is to inhibit the uptake of exosomes by recipient cells. Heparan sulfate proteoglycans (HSPGs) are internalizing receptors of cancer-derived exosomes. Internalized exosomes colocalized with HSPGs of the syndecan and glypican type, enzymatic depletion of cell surface HSPG significantly attenuates exosome uptake [78]. Consistently, mouse fibroblasts isolated from syntenin knockout mice show lesser amounts of HSPGs, which correlates with reduced uptake of exosomes, suggesting that syntenin may be a nonnegligible target for inhibiting tumor development [79]. The normal pancreatic tissue around PDAC tissue release REG3β, a glycoprotein-binding lectin on the exosome surface that interferes with their uptake and internalization by target cells. In vivo, tumor cells significantly impair the uptake of REG3β+ exosomes, thereby inhibiting the migration of pancreatic cancer cells [80]. The above evidence indicates that target exosome secretion and uptake is a promising method to treat pancreatic cancer.

### 5.2. Exosomes as Nanotransporters

Exosomes can carry various nucleic acids and proteins to be ingested by recipient cells, which can be used as ideal drug carriers due to their biological characteristics. Drugs with toxicity or immunogenicity can be encapsulated in exosomes and transferred to target cells to avoid their systemic toxicity. MSCs can package and deliver paclitaxel (PTX) through exosomes, which can significantly inhibit the proliferation of pancreatic cancer cell lines [81]. Kim et al. also confirmed that the addition of PTX-loaded exosomes in drug-resistant cells could increase cytotoxicity by more than 50 times. The PTX-loaded exosomes have great potential for delivery of chemotherapeutic drugs and treatment of drug-resistant cancers [82]. Simultaneously, exosomes have also been used to deliver functional nucleic acids and proteins, with the aim to avoid side effects and enhance targeting efficacy. Recent study has shown that purified exosomes from HEK293 cells are loaded with exogenous siRNA using ultrasound treatment or electroporation, and the growth of tumor cells is inhibited by silencing the HER2 gene [83]. Oncogenic Kras^G12D^ is a common mutation in PC, and by loading KRAS ^G12D^ siRNA into exosomes, mouse models of pancreatic cancer were suppressed and overall survival profoundly improved [84]. The mature development of Clustered Regularly Interspaced Short Palindromic Repeats (CRISPR)/Cas9 makes it an effective tool for gene editing and a great prospect for treatment. A safe and effective intracellular delivery system is critical to its application as a therapeutic genome editing technique. Due to low tolerance and immunogenicity, lentiviruses, plasmids and other vectors carrying CRISPR/Cas9 limit their delivery in vivo, while the exosomes make it possible to encapsulate the CRISPR/Cas9 gene editing system. Chen et al. found that there is potential for endogenous exosomes to be used as safe and effective delivery vectors for functional gRNA and Cas9 proteins. Meanwhile, the endogenous exosome-mediated gene editing system can be delivered to cells or tissues, and a single guiding RNA (gRNA) and Cas9 protein can be loaded into endogenous exosomes independently and taken up by recipient cells [85]. Hybrid exosomes can be formed by co-incubation of exosomes with liposomes. This hybrid exosome has the ability to encapsulate macromolecular proteins and nucleic acids, including the CRISPR/Cas9 expression vectors. Furthermore, the hybrid exosomes can be uptake by MSCs that cannot be transfected by liposome and exert gene editing effect. [86].

## 6. Limitations and Future Perspectives

Since the first discovery of exosomes, there has been an accumulation of knowledge of the mechanisms of exosome formation, secretion and uptake, and a unified consensus on exosome isolation and identification. Due to the complexity and special biological properties of exosomes, the design of a method to isolate exosomes in a simple, rapid and sensitive manner is still the barrier limiting their clinical application. Although a series of exosome biomarkers with great potential of early diagnosis and prognosis of pancreatic cancer have been described, a more rapid and economical method for isolation and purification of exosomes is required for their application in clinical practice. More importantly, most studies have been conducted with a relatively small number of samples, and larger clinical trials are still needed to verify its efficacy. Current research on exosomes as a treatment method for pancreatic cancer is mainly based on cell lines and animal models. How exosomes can be used to transfer drugs in a safe and effective way, and how to achieve in vivo gene editing, remain problems to be solved.

With the development of precision medicine, exosomes have become promising biomarkers and valuable therapeutic targets. Despite these limitations, exosomes have great promise in the diagnosis and treatment of multiple types of human malignancies. A series of recent phase I and II clinical trials have confirmed that exosomes play a potential role as drug carriers and cell-free vaccines in anticancer therapy. The autologous dendritic cell (DC)-derived exosome (DEX) loaded with tumor antigen can promote the anticancer immune response in patients with advanced melanoma and advanced non-small cell lung cancer (NSCLC) [87,88]. Further studies have shown that exosomes derived from IFN-maturated DCs stimulate the activation of NK cells and improve the rate of progression-free survival in unresectable NSCLC patients [89]. These clinical trials provided alternative strategies for exosome-based anti-cancer therapies, and confirmed the feasibility of large-scale clinical exosome production and the safety and effectiveness of the DEX vaccine in treating patients with advanced cancers. It is believed that there will be clinical trials based on this method in patients with advanced pancreatic cancer in the near future.

## 7. Conclusions

Exosomes secreted from pancreatic cancer cells and their surrounding related cells can communicate with each other by binding with the corresponding receptors on the cell membrane, which leads to the heterogeneity of tumors, and changes in tumor-related cells and the microenvironment. As mentioned above, exosomes are involved in various pathological processes, and microenvironment remodeling, and are vital in promoting tumorigenesis, proliferation, and metastasis. Current clinical research has concentrated on the advancement of exosomes as indicators for early detection and prognosis. Regardless of the fact that the research progress on exosomes is exciting, many problems still need to be further clarified. For example, it is difficult to isolate and purify exosomes owing to their content and structure, and new methods should be proposed to improve their yield and purity. In addition, the cases included in relevant clinical studies are not enough, and the clinical application of exosomes in the differential diagnosis of pancreatic cancer needs further large-scale cohort studies to be performed. Despite the increasing research on exosomes, the effects of exosomes on the response to chemotherapy and radiotherapy are less well studied, and further research and exploration of their molecular mechanisms are still required. Biological characteristics of exosomes determine their potential in signaling and drug delivery. Currently, exosomes have served as drug carriers for targeted tumor treatment. However, there is still a long way to go in developing applications for exosomes in anti-tumor therapy.

## Figures and Tables

**Table 1:** Functions of exosomes in pancreatic ductal adenocarcinoma (PDAC).

Specific Role	Origin of Exosomes	Function in PDAC	References
**Proliferation**	gemcitabine-treated CAFs	increase proliferation increased level of Snail and miR-146a in recipient cells	[45]
	BMSC	over-expressed exosomal miR-126-3p inhibits the proliferation by targeting down-regulation of ADAM9	[46]
	highly malignant PC cells	upregulate exosomal protein ZIP4 in moderate malignant PC and enhance their ability to proliferate	[47]
	TAS	inhibit proliferation on adjacent PDAC cells via the delivery of miR-145	[48]
**Invasion and Metastasis**	hypoxic PC cells	upregulate miR-301a-3p to enhance metastatic capacity	[50]
	PC cells	exosomal miR-222 activates AKT by inhibiting PPP2R2A expression to promote invasion and metastasis	[51]
**Chemoresistance**	CAFs	increase the release of Snail and miR-146a via exosomes and confer chemoresistance	[45]
	CAFs	exosomal miR-21 induces chemoresistance by activating PI3K/AKT signaling pathway or binding APAF1	[63]
	PC cells	exosomeal miR-155 promotes chemoresistance through the anti-apoptotic pathway and inhibits dCK	[16,64]
	PC cells	confer chemoresistance by enhancing the detoxification of ROS through the expression of SOD2 and CAT	[16]
	chemoresistant PC cells	increase the gemcitabine resistance via transferring EphA2 to less chemoresistant PC cells	[65]

**Abbreviations:** cancer-related fibroblasts, CAFs; bone marrow mesenchymal stem cell, BMSC; a disintegrin and a metalloproteinase-9, ADAM9; tumor-associated stroma, TAS; apoptotic peptidase activating factor 1, APAF1; deoxycytidine kinase, dCK; superoxide dismutase 2, SOD2; ephrin type-A receptor 2, EphA2.

**Table 2:** Exosomes as biomarkers in PDAC.

Biomarkers	Sample	Clinical Significance	References
miR-17-5p, miR-21	22 PCs, 6 benign pancreatic tumors, 7 ampullary carcinomas, 6 CPs, 8 healthy control	Diagnostic biomarker for dividing PC and non-PC	[68]
miR-10b	3 PDACs, 3 CPs, 3 healthy control	Diagnostic biomarker for PDAC comparing with CP and normal control	[70]
miR-196a, miR-1246	15 PDACs (Stage I-IIA), 15 healthy control	Diagnostic biomarker for dividing PDAC and non-PC	[72]
miR-451a	7 PDACs with stage I, 43 PDACs with stage II, 20 healthy control	Predicting biomarker for recurrence and survival	[73]
Glypican-1	32 breast cancer, 190 PDACs, 100 normal control	Diagnostic biomarker for dividing PC and benign pancreatic disease, prediction of prognosis	[37]
migration inhibitory factor	40 PDACs, 15 healthy control	Prognostic marker for PDAC liver metastasis	[75]
exosomal integrin	27 PDACs, 13 healthy control	Capable of predicting progression of cancer	[76]

**Abbreviations:** pancreatic cancer, PC; pancreatic ductal adenocarcinoma, PDAC; chronic pancreatitis, CP.

## Abbreviations

CAFs	cancer-related fibroblasts
dCK	deoxycytidine kinase
ECM	extracellular matrix
EMT	epithelial-mesenchymal transition
EVs	extracellular vesicles
MSCs	mesenchymal stem cells
MVBs	multivesicular bodies
PC	pancreatic cacer
PDAC	pancreatic ductal adenocarcinoma
UC	ultracentrifugation
TEM	transmission electron microscopy
